# Concomitant gastric carcinoma and primary hepatic angiosarcoma in a patient: A case report

**DOI:** 10.1515/biol-2022-0961

**Published:** 2025-11-29

**Authors:** Qi Wang, Xuehai Xie, Yinmo Yang, Xiaodong Tian

**Affiliations:** Hepatopancreatobiliary Surgery Department, Peking University First Hospital, 8 Xishiku Street, Xicheng District, Beijing, 100034, China

**Keywords:** gastric cancer, primary hepatic angiosarcoma, multiple primary cancer

## Abstract

Primary hepatic angiosarcoma (PHA) is a rare malignancy, and the occurrence of a dual malignancy involving both gastric cancer (GC) and PHA is even more exceptional. Here, we present a case of a 63-year-old man who complained of hiccups. PET-CT and Abdomen & Pelvis Routine Enhanced Scan results revealed a single liver mass and thickening of the inner wall of the gastric antrum. The pathologic biopsy confirmed the presence of GC in the antrum along the greater curvature. The initial diagnosis indicated GC with solitary liver metastasis, given the rarity of PHA. The patient underwent a radical distal gastrectomy and right posterior hepatic lobectomy. Postoperative pathology revealed early GC and PHA without any signs of lymphatic or distant metastases.

## 1 Introduction

Gastric cancer (GC) is a prevalent malignant tumor that originates from epithelial cells in the stomach, ranking third in both incidence and mortality rates in China [1]. For early gastric cancer (EGC) patients who undergo endoscopic submucosal dissection (ESD), the 5-year rates of overall survival (OS), disease-specific survival, and relative survival were 92.6, 99.9, and 105.0%, respectively [2]. However, EGC with lymph node metastases has a relatively poor prognosis even after standard surgery [3]. Comprehensive therapies, including adjuvant and palliative chemotherapy, could be considered for these patients as well as those with advanced stages. Despite advancements in understanding the biological aspects of GC, surgical dissection or ESD remains a crucial cornerstone in the treatment with curative intent [4,5].

Primary hepatic angiosarcoma (PHA) is a rare and highly aggressive neoplasm that originates from the endothelial cells of blood vessels or lymphatic vessels. Approximately 200 cases are diagnosed annually worldwide [6]. Epidemiological prospective research indicates an incidence rate of PHA at approximately 0.5–2.5 cases per 10 million individuals [7]. Despite its rarity, it is the most common primary malignant mesenchymal tumor of the liver [6]. PHA is associated with prolonged latency following exposure to known chemical carcinogens such as vinyl chloride; however, 75% of the patients with no identifiable etiology [6,8]. Typically, PHA manifests in older men, with the highest incidence occurring in the sixth decade of life, and males are more commonly affected than females, with a ratio of 3–4:1 [6]. PHA is a rapid fatal neoplasm, with most patients succumbing within 6 months due to hepatic insufficiency or hemorrhagic complications [9,10]. Only 3% of the patients live more than 2 years with treatment [11], and the life expectancy after a liver transplant is less than 7 months [12]. Therefore, early diagnosis and radical resection of the tumor are crucial for effective treatment.

Multiple primary cancer (MPC) refers to the presence of more than one distinct malignant tumor diagnosed in a single patient, either concurrently or consecutively [13]. According to the criteria proposed by the World Health Organization in 2005 [14]: 1. the malignancy of each tumor must be confirmed through pathological examination; 2. each tumor must exhibit unique pathological morphology; 3. the tumors must occur at different sites; and 4. tumor metastasis must be excluded. Here, we present a case in which the patient was diagnosed with concurrent GC and HPA. Our aim is to provide evidence supporting surgical intervention as the optimal approach for early PHA. Additionally, we emphasize the importance of prioritizing liver puncture to identify the nature of digestive system tumors with a single hepatic mass. Such patients should also be given priority for surgical treatment rather than preoperative neoadjuvant therapy.

## 2 Case report

**Figure 1:**
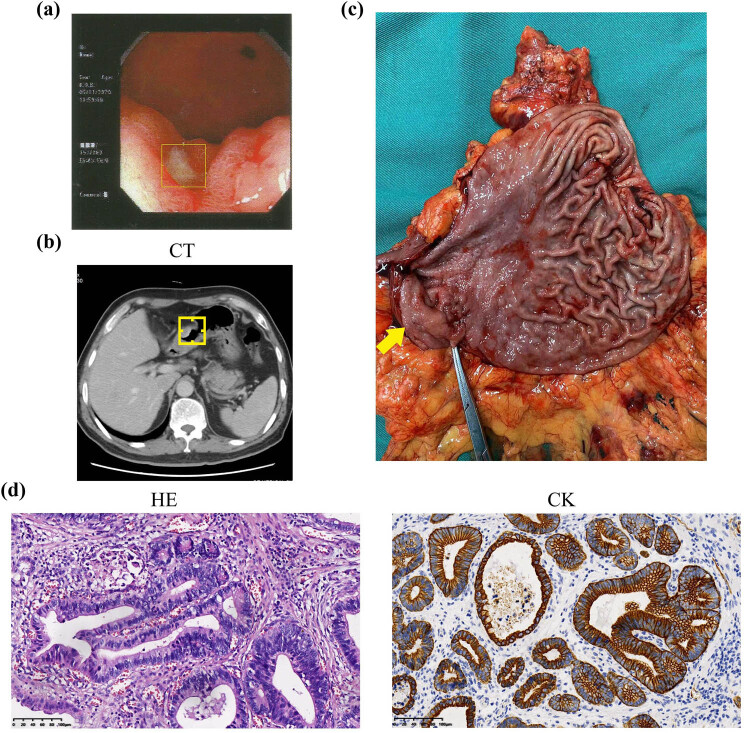
(a) The endoscopic biopsies confirmed the presence of gastric antrum cancer accompanied by mild chronic atrophic gastritis (C-2type) in the lesion located around the great curvature side of the antrum; (b) the Abdomen & Pelvis Routine Enhanced Scan CT detected the presence of a gastric lesion in the patient; (c) the postoperative specimen of GC; (d) the histopathological findings, encompassing hematoxylin and eosin (HE) staining and CK immunohistochemistry, unequivocally confirmed the presence of GC in the gastric lesion.

A 63-year-old male patient, who had a habit of consuming pickled foods, was admitted to the hospital due to persistent hiccups lasting for over 6 months. A gastroscopic examination conducted at a local hospital revealed an off-white lesion measuring 2.0 cm × 2.0 cm in the antrum of the stomach (Figure 1a). Pathologic examination revealed the presence of high-grade dysplasia and intramucosal adenocarcinoma. Subsequently, the patient was referred to Peking University First Hospital for surgery.

**Figure 2:**
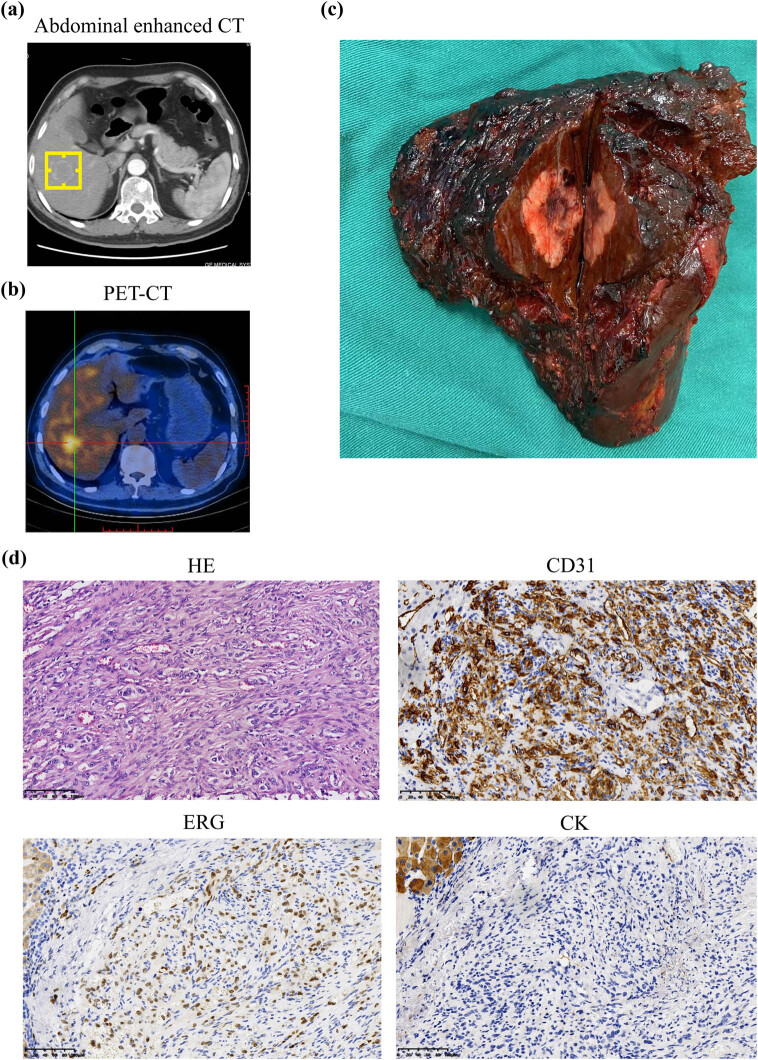
(a) The Abdomen & Pelvis Routine Enhanced Scan CT revealed a solitary hepatic lesion; (b) the PET-CT scan confirmed elevated glucose metabolism in the hepatic lesion; (c) the liver specimen; (d) the histopathological findings encompassing HE staining, CD31 immunohistochemistry, ERG immunostaining, and CK immunohistochemistry, unequivocally confirmed the presence of hepatic angiosarcoma in the liver specimen.

The physical assessment showed no notable abnormalities, and tumor markers, including alpha-fetoprotein (AFP), carcinoembryonic antigen, carbohydrate antigen 19-9, total prostate-specific antigen (tPSA), free prostate-specific antigen (fPSA), fPSA/tPSA (f/t), squamous cell carcinoma associated antigen, cytokeratin 19 fragments (CYFRA21-1), neuron-specific enolase, carbohydrate antigen 72-4, pro-gastrin-releasing peptide, and carbohydrate antigen 24-2, were within normal range. The Abdomen & Pelvis Routine Enhanced CT scan revealed thickening of the inner wall of the gastric antrum (Figure 1b). Additionally, the enhanced CT scan showed a solitary mass in the liver with ring-like enhancement at the periphery in the arterial phase, indistinct boundaries during the venous phase, and ring-like enhancement at the edges in the subsequent delayed phase (Figure 2a). The solitary hepatic mass was initially identified as a metastatic lesion originating from GC, consistent with the liver being the primary site of metastasis for tumors originating from the digestive system. PET-CT, used for detecting metastases, showed elevated glucose metabolism in the hepatic lesion (Figure 2b).

A successful radical distal gastrectomy and resection of the right posterior lobe of the liver were performed, as surgery is the optimal therapeutic approach for this patient [15]. Following the operation, the hiccup symptom was significantly relieved. The gastric lesion, measuring 22 mm × 20 mm × 5 mm and accompanied by a superficial ulcer (Figure 1c), was diagnosed as early-stage gastric cancer (T_1b_N_0_M_0_) (Figure 1d). Immunohistochemical analysis of the 33 mm × 25 mm × 25 mm mass in the right liver (Figure 2c) demonstrated positive expression of CD31 [6], ERG [16], and negative expression of CK markers (Figure 2d), leading to a diagnosis of PHA. Fortunately, the patient’s GC and PHA showed no evidence of lymph node metastases or distant metastases. The patient recovered well and was discharged from the hospital. He was advised to undergo a review of tumor markers, and abdominal CT scans every 3 months. At 36-month follow-up, he remained well and disease-free with no subsequent need for chemotherapy.


**Informed consent:** Informed consent has been obtained from all individuals included in this study.
**Ethical approval:** The research related to human use has complied with all the relevant national regulations and institutional policies, is in accordance with the tenets of the Helsinki Declaration, and has been approved by the Peking University First Hospital (2024 study 194-002).

## 3 Discussion

This special case presents two distinctive characteristics. First, PHA, an aggressive and rare tumor, poses a challenge due to the lack of established and effective treatment guidelines owing to its low frequency [6]. Second, the concurrent EGC adds to the rarity of this case.

In this case, the patient initially presented with gastrointestinal symptoms and a solitary mass in the liver was detected during a CT scan. Given the rarity of PHA, the negative of AFP, and the liver being identified as the primary site of metastasis for GC [17], our initial diagnosis for the patient was primary GC with solitary hepatic metastasis. In light of this, the patient was presented with two options: neoadjuvant chemotherapy followed by surgical intervention or direct progression to surgery. In recent decades, there has been an evaluation of comprehensive therapy methods involving a combination of surgery, chemotherapy, and gastrectomy with trans-arterial chemoembolization plus systemic chemotherapy (GTC) in patients with GC and liver metastasis [18]. Some patients are ineligible for surgical procedures due to the association of liver metastasis with extrahepatic diseases, such as peritoneal dissemination, lymph node metastasis, and direct invasion of other organs by cancer [19]. However, Saiura et al. [20] argued that patients with liver metastasis from GC who undergo hepatic resection have a higher likelihood of long-term survival in the absence of lymph node metastasis at the primary site. Markar et al. [15] found that among patients diagnosed with GC, those who underwent curative surgery combined with hepatectomy had a better survival time compared to those who only underwent gastrectomy, and their survival time was similar to that of patients undergoing gastrectomy without liver metastasis. Other studies have also suggested that surgical resection of liver metastases from GC may benefit patients with solitary metastasis [21,22,23]. The successful surgical procedure involved distal gastrectomy and resection of the right posterior lobe of the liver. This decision was prompted by the presence of a solitary liver mass and the absence of metastases on PET-CT. Subsequent pathological analysis confirmed that the liver lesion is PHA based on the immunohistochemistry evidence and immune markers.

PHA typically presents as a multicentric occurrence involving both lobes of the liver, with multifocal masses occasionally affecting the entire hepatic tissue [6]. Abdominal pain was the most common symptom for 66% of the patients, while 12.5% of the patients presented with atypical symptoms [16]. Patients may also present with ascites, heart palpitations, cough, hepatomegaly, acute abdominal crisis, backache, weakness, weight loss, fever, anemia, hemoperitoneum, and other symptoms indicative of distant metastases (such as lung, peritoneum, bone, and spleen) [6,9,11,16]. In this study, the primary symptom in this patient was persistent hiccups, not hepatic discomfort, despite the presence of a large mass in the liver. Due to the absence of specific imaging features, pathological biopsy remains the primary diagnostic method. However, fine needle aspiration cytology is limited by the potential risk of fatal hemorrhage [24]. CD31 [6] and ERG [16] have been proven to be sensitive and specific diagnostic markers. Vimentin, GPC-3, desmin, Ki-67, CD34, and factor VIII-related antigens have been reported as positive in PHA [6,16]. The pathological findings in this case were consistent with the aforementioned markers. It is worth noting that while the cytokeratin expression of PHA in this case is negative, some angiosarcomas have been shown to be positive for cytokeratin [25,26]. In the absence of CD31 and ERG expression, a positive cytokeratin result may lead to the misdiagnosis of the tumor as epithelial in origin.

Radical surgical resection appears to be the most effective treatment method for PHA [10]. Due to the lack of noticeable symptoms and the aggressive nature of PHA, only 20% of the cases are considered suitable for hepatic resection [6]. Liver transplantation appears to be an effective treatment option for patients with entire liver involvement by tumors. However, certain studies have cautioned against performing liver transplantation in patients with PHA due to the high recurrence rate and rapid disease progression, which leads to a median survival of less than 7 months post-transplantation [12,27]. Kim et al. [28] demonstrated that chemotherapy improved survival rates in 2 out of 4 patients with advanced PHA and distant metastasis. The study recommended the utilization of 5-FU-carboplatin in combination with doxorubicin or ifosfamide for treatment. Palliative chemotherapy may be the only treatment option available for those patients. A study showed that the median OS and disease-free survival of patients with PHA after surgical resection were 18 months (range: 3–144 months) and 10 months (range: 2–144 months), respectively [29]. In a study involving six patients diagnosed with PHA and presenting with a solitary mass, three patients underwent right hepatectomy, one patient underwent extended right hepatectomy, and two patients underwent left hepatectomy. Among these patients, two survived for more than 12 months post-surgery, and one patient achieved a survival period of over 29 months without recurrence [30]. In this case, although the PHA volume measured 33 mm × 25 mm × 25 mm, postoperative pathology revealed no signs of lymph node metastasis, and PET-CT indicated no distant metastasis. The patient did not receive chemotherapy following surgery but was advised to undergo a review every 3 months. Presently, it has been 36 months since the surgery, and no recurrence has been observed. This case helps bolster our support for surgery as the optimal treatment for PHA, capable of extending patients’ survival.

In this case, whether it was GC or PHA, the patient did not exhibit typical symptoms. GC is currently believed to be primarily caused by *H. pylori* infection, autoimmune atrophic gastritis, and poor dietary habits, while PHA is primarily associated with exposure to known chemical carcinogens over a prolonged period, although 75% of the patients have no known etiology [6]. The etiologies of the two conditions do not significantly overlap. Furthermore, there have been no previous reports, to the best of our knowledge, of both occurring simultaneously or sequentially in a single patient. Genetic testing was conducted for the patient’s two tumors. In the case of GC, we detected 0 primary mutations, 1 secondary mutation (VEGFA), and 7 tertiary mutations (ASXL1, CARD11, CDKN2A, E2F3, GRIN2A, PIK3CA, and TP53). Interestingly, the above-mentioned mutated genes were not detected in the PHA. This indicates that the patient’s GC and PHA are not directly related. Additionally, both PHA and GC are MSS/MSI-L.

In summary, it is important to carefully consider the potential presence of PHA in AFP-free cases where an endoscopic biopsy indicates EGC and a CT scan reveals a hepatic mass. While standardized treatment guidelines for PHA are still being developed, surgical intervention remains the optimal choice for patients with a solitary hepatic mass. In the absence of a lymph node or distant metastatic PHA, chemotherapy may not be necessary. Additionally, we recommend prioritizing surgical treatment for patients with GC accompanied by a solitary liver mass, regardless of whether the liver mass is a metastasis.
